# Met Receptor Acts Uniquely for Survival and Morphogenesis of EGFR-Dependent Normal Mammary Epithelial and Cancer Cells

**DOI:** 10.1371/journal.pone.0044982

**Published:** 2012-09-13

**Authors:** Paolo Accornero, Silvia Miretti, Francesca Bersani, Elena Quaglino, Eugenio Martignani, Mario Baratta

**Affiliations:** 1 Department of Veterinary Science, University of Torino, Grugliasco (TO), Italy; 2 Department of Oncology and Center for Experimental Research and Medical Studies, University of Torino, Torino, Italy; 3 Molecular Biotechnology Center, Department of Clinical and Biological Sciences, University of Torino, Torino, Italy; University of Central Florida, United States of America

## Abstract

Mammary gland development and breast cancer growth require multiple factors both of endocrine and paracrine origin. We analyzed the roles of Epidermal Growth Factor Receptor (EGFR) and Hepatocyte Growth Factor Receptor (Met) in mammary epithelial cells and mammary tumor cells derived from a mutated-ErbB2 transgenic mice. By using highly specific tyrosine kinase inhibitors we found that MCF-10A and NMuMG mammary epithelial cell lines are totally dependent on EGFR activation for their growth and survival. Proliferation and 3D-morphogenesis assays showed that HGF had no role in maintaining mammary cell viability, but was the only cytokine able to rescue EGFR-inhibited mammary cells. Insulin-Like Growth Factor-I (IGF-I), basic-Fibroblast Growth Factor (b-FGF) and Neuregulin, which are well known mammary morphogenic factors, did not rescue proliferation or morphogenesis in these cell lines, following EGFR inhibition. Similarly, ErbB2-driven tumor cells are EGFR-dependent and also display HGF-mediated rescue. Western-blot analysis of the signaling pathways involved in rescue after EGFR inhibition indicated that concomitant ERK1/2 and AKT activation was exclusively driven by Met, but not by IGF-I or b-FGF. These results describe a unique role for EGFR and Met in mammary epithelial cells by showing that similar pathways can be used by tumorigenic cells to sustain growth and resist to EGFR-directed anti-tumorigenic drugs.

## Introduction

Circulating hormones like estrogen, progesterone, growth hormone and prolactin were among the first endocrine factors which were identified to be necessary for mammary gland morphogenesis and differentiation during growth, reproductive cyclicity and pregnancy [1]. Although a direct role for these hormones is known, recent evidences show that their main biological activities are achieved through the release of local growth factors by mammary epithelial and stromal cells. These factors subsequently diffuse and activate their respective receptors in the stromal or epithelial compartments promoting an epithelial-mesenchymal interaction. Both cellular compartments of the gland are thus required for the correct development of this organ [2]–[4]. These indirect signaling mechanisms ensure that the systemic stimulus is amplified within the target organ and that different cell types participate in the morphogenic events in a coordinated manner and fine-tuned according to local requirements.

Most of these locally released molecules act through specific tyrosine kinase receptors (RTK) promoting several biological responses, like proliferation, remodelling of the extracellular matrix and motility of the target cells. Although some of these factors have a precise and unique role during morphogenesis or remodelling of the gland, many signaling pathways activated downstream of different RTKs are identical. Thus, these pathways may act as redundant when activated simultaneously in the same cell type, possibly reinforcing the phosphorylation cascade and its final biological effect.

One of the best described RTK that act as a fundamental morphogenic modulator of the mammary gland is the Epidermal Growth Factor Receptor (EGFR). Within the rodent mammary gland, locally released amphiregulin, whose only known receptor is EGFR, was found to mediate estrogen signaling during pubertal mammary development. The steroid hormone acts by stimulating amphiregulin release by the estrogen receptor positive epithelial compartment of the gland. Amphiregulin then promotes EGFR activation within the stromal compartment of the gland driving the correct branching of this organ [5], [6]. Although the main targets of amphiregulin are stromal cells, this does not rule out that EGFR signaling also has a role in the epithelium. EGFR is expressed in both stromal and epithelial compartments [7], [8], and other EGFR ligands, in particular EGF, are highly expressed in the glands during pregnancy [9]. Thus our first aim was to evaluate whether EGFR plays a role in mammary epithelial cell growth and turnover. We did this by targeting this receptor with highly specific inhibitors. The difficulty of clarifying the role of EGFR in the adult mammary epithelial compartment is possibly due to the fact that other receptors, with a similar expression pattern, may substitute for the absence of EGFR or its ligands *in vivo*. In fact, stromal fibroblasts produce many signals to the neighbouring epithelial cells. Several growth factors, such as Insulin-like Growth Factor (IGF), Fibroblast Growth Factor (FGF), Neuregulin and Hepatocyte Growth Factor (HGF) are expressed in the mammary stroma, whereas their respective receptors are found in the epithelium [1]. Specifically, IGF-I has a major role in ductal morphogenesis, and may be necessary for later stages of mammary development [10]. FGFR2 is involved in mammary development, in particular in proliferation and invasion by terminal end buds, but seems dispensable in the mature gland [11], [12]. Neuregulin operates during lobulo-alveolar development at pregnancy [13], [14]. Finally, the tyrosine kinase receptor for HGF, Met, was found to play a role in mammary branching morphogenesis in *ex vivo* and *in vitro* experiments [15], [16]. Met could be a good candidate for EGFR replacement since recent evidence has shown that this receptor and EGFR can act cooperatively during kidney development to regulate ureteric bud branching and mediate maintenance of the normal adult collecting duct [17].

In this study we evaluated in mammary epithelial cell lines whether other receptors, usually present in the mammary gland, could sustain similar EGFR-like activities and signal transduction pathways when EGFR signaling was ablated by administration of highly specific inhibitors. Finally, we tested if such compensatory mechanisms were also present in tumor cells isolated from a well described transgenic mouse model of ErbB2 mammary tumorigenesis.

## Results

### Mammary Epithelial Cells Express EGFR and Met Receptors and Respond to HGF or EGF Treatment with Phosphorylation of their Respective Receptors and Activation of the ERK1/2 and AKT Pathways

**Figure 1:**
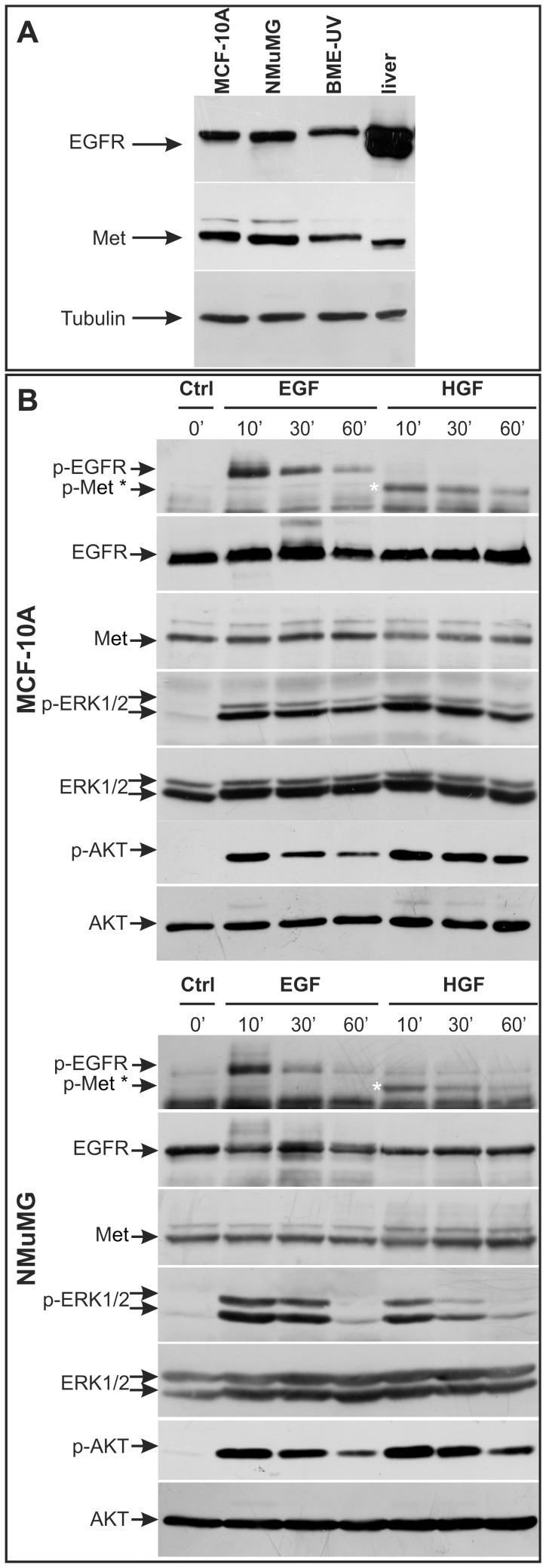
Expression and activation of EGFR and Met in mammary epithelial cells. A. Western-blot analysis of EGFR and Met expression in human MCF-10A, murine NMuMG and bovine BME-UV mammary epithelial cells. Mouse liver extract was used as a positive control for both Met and EGFR. **B.** Western-blot analysis of EGFR, Met, ERK1/2 and AKT phosphorylation in MCF-10A and NMuMG cells cultivated in serum-starving medium and treated with EGF or HGF (20 ng/ml) for 10, 30 and 60 min. EGFR, Met, ERK1/2 and AKT were used to show comparable loadings between the lanes.

We first analyzed the expression of EGFR and Met receptors and the biochemical response to their respective ligands in 3 mammary epithelial cell lines of different origin. All cells expressed Met and EGFR (Fig. 1A). MCF-10A and NMuMG stimulated with HGF or EGF for 10 min, 30 min or 60 min displayed an increase in phospho-EGFR, phospho-Met, phospho-ERK1/2 and phospho-AKT levels that gradually returned close to basal levels (Fig. 1B). Similar data have been already described for the BME-UV cell line [18].

### EGFR is Required for Viability of a Subset of Mammary Epithelial Cell Lines, while Met Activation is Dispensable

To test whether EGFR or Met activity modulate viability in the mammary cell lines we used highly specific receptor tyrosine kinase inhibitors (RTKi; AG1478 for EGFR and PHA-665752 for Met) at nanomolar concentrations. We first tested the specificity of these inhibitors by stimulating serum-starved MCF-10A cells with HGF or EGF together with AG1478 or PHA-665752 at 250 nM (Fig. S1). Western blot analysis showed that AG1478 and PHA-665752 inhibited exclusively the phosphorylation of EGFR and Met respectively.

**Figure 2:**
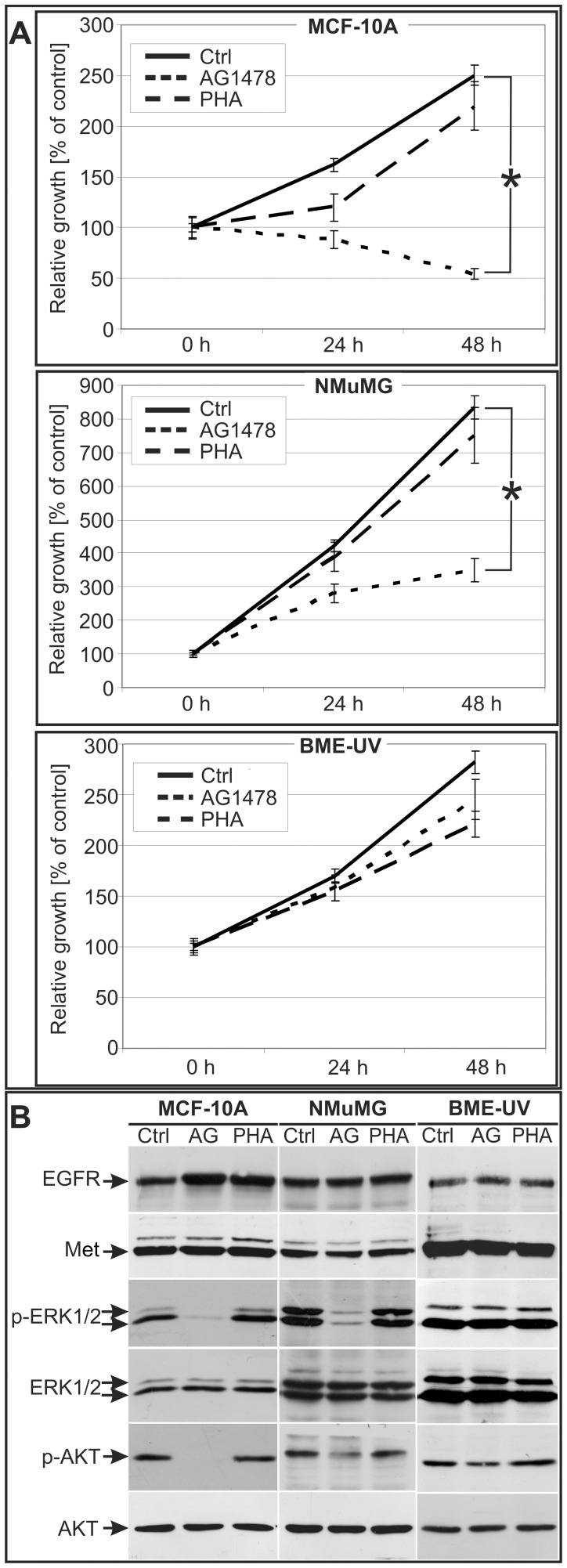
MCF-10A and NMuMG mammary epithelial cells show EGFR dependence. A: MTT assay of MCF-10A, NMuMG and BME-UV cells at 0 h, 24 h and 48 h. Cells were cultivated in their growth medium (see materials and methods) and either left untreated (Ctrl) or treated with PHA-665752 (PHA) or AG1478 (both 250 nM). MTT value at 0 h was set to 100%. Columns, mean (n = 6); bars, S.E.M. *P<0.01. **B.** Western-blot analysis of ERK1/2 and AKT phosphorylation in mammary cells cultivated in their respective growth medium without (Ctrl) or with the RTK inhibitors (250 nM, 1 h) PHA-665752 (PHA) or AG1478 (AG). EGFR, Met, ERK1/2 and AKT were used to show comparable loadings between the lanes.

These inhibitors were then used in a viability assay to test the dependence of different mammary cell lines on Met or EGFR signaling (Fig. 2A). Cells, cultured in their respective growth medium, were treated with AG1478 and PHA-665752 (250 nM) and monitored by MTT at 24 and 48 h. All mammary epithelial cell lines were unaffected by Met inhibitor PHA-665752. EGFR inhibition by AG1478, did not modify BME-UV viability, while MCF-10A and NMuMG cell viability was greatly impaired.

To determine the mechanism of viability blockade mediated by EGFR inhibition we assayed the activation state of ERK1/2 and AKT following AG1478 or PHA-665752 treatment in cells cultured in proliferation medium (Fig. 2B). In response to treatment with AG1478, but not PHA-665752, MCF-10A and NMuMG cells showed a reduction of both ERK1/2 and AKT phosphorylation. In agreement with the inability of the inhibitors to reduce viability, ERK1/2 and AKT activation states were unaltered under all conditions in BME-UV cells.

### The HGF-Met Axis Restores Proliferation, Survival and Morphogenesis in NMuMG and MCF-10A Cells Deprived of EGFR Activity

**Figure 3:**
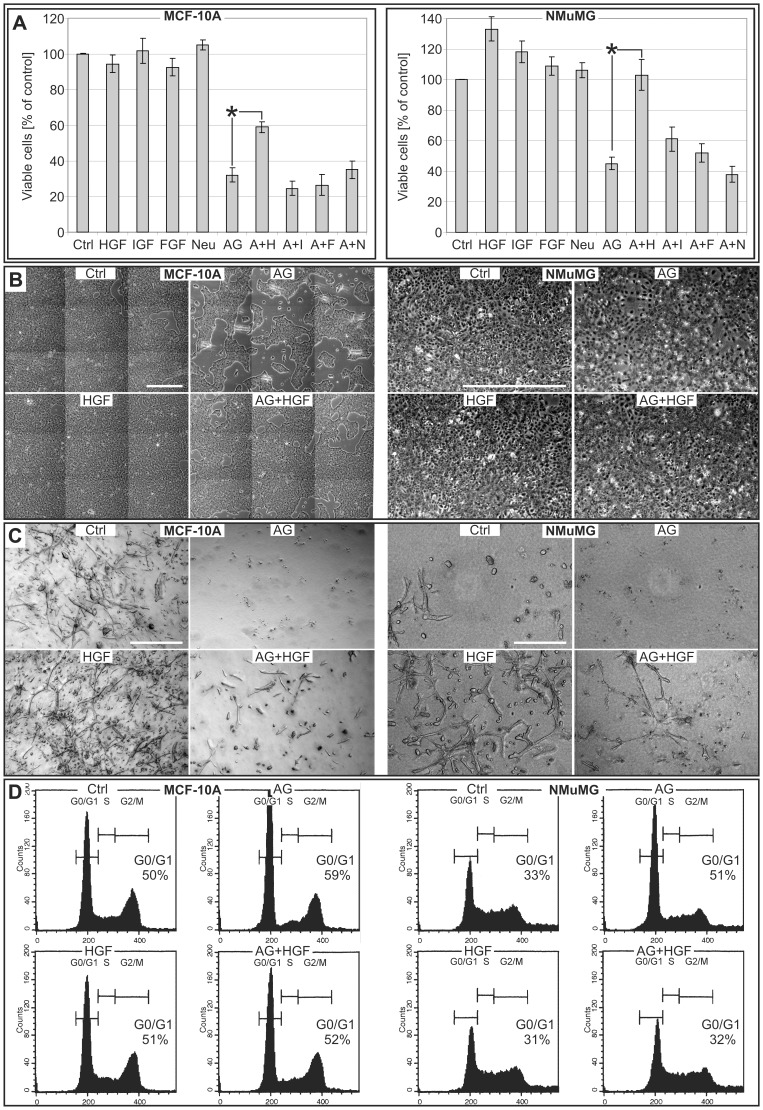
HGF act as a recovery agent in EGFR inhibited mammary epithelial cells. A. Viable cell count by trypan-blue exclusion staining at 48 h of MCF-10A (left panel) and NMuMG (right panel) cells cultivated in their respective growth medium and treated with the indicated factors (HGF 10 ng/ml (H), IGF-I 100 ng/ml (I), b-FGF 20 ng/ml (F), Neuregulin 20 ng/ml (N); AG1478 250 nM (A)). Untreated control (Ctrl) was set to 100%. Columns, mean (n = 6); bars, S.E.M. *P<0.05. **B.** MCF-10A cells (left panels; composite of 9 fields) and NMuMG cells (right panels) cultivated in their respective growth medium for 24 h with the indicated factors (HGF 10 ng/ml; AG1478 250 nM). Bar  = 1000 µm. **C.** Extended death of field composite image obtained from multiple Z-stacking pictures of MCF-10A cells (left panels) and NMuMG cells (right panels) grown in collagen for 4 days with the indicated factors (HGF 10 ng/ml; AG1478 250 nM). Bar  = 500 µm. **D.** Cell cycle distribution examined by propidium iodide staining and FACS analysis of MCF-10A cells (left panels; 8 h) and NMuMG cells (right panels; 16 h) cultivated in growth medium and treated with the indicated factors (HGF 10 ng/ml, AG1478 250 nM).

We next examined whether Met activation could rescue cells from growth impairment following EGFR inhibition. HGF acted as a recovery agent in MCF-10A and NMuMG cells treated with AG1478 (Fig. 3A). Since IGF-I, b-FGF and Neuregulin have been described as important mediators of mammary development [10], [11], [14] we tested their effect as possible alternative recovery agents in the same cell lines. Both did not increase proliferation in response to these growth factors following AG1478 treatment (Fig. 3A). HGF mediated recovery in cells treated with AG1478 was evident as increased confluence in MCF-10A (that grow as compact islands; Fig. 3B left panels) and increased cell density in NMuMG cells (that grow as dispersed cells; Fig. 3B right panels).

NMuMG and MCF-10A cell lines possess morphogenic capabilities when resuspended and cultured in a 3-D matrix like collagen [16], [19]. AG1478 had a dramatic effect on 3D-morphogenesis of both cell lines, inducing death of all cells (Fig. 3C, AG and Video S1). HGF was the only tested growth factor able to recover cells from EGFR inhibition (Fig. 3C, AG+HGF and Video S2). EGF, IGF-I, b-FGF and Neuregulin were unable to recover cell death following AG1478 treatment (not shown). Met specificity to HGF recovery in MCF-10A and NMuMG cells was confirmed using the highly specific Met inhibitor PHA-665752 at 250 nM together with AG and HGF. In the presence of this RTKi, HGF lost its capacity to recover mammary cells from EGFR inhibition (Fig. S2, A+P+H).

Cells cycle analysis by FACS showed that EGFR inhibition led to an increase in the percentage of cells in G0/G1 phase (Fig. 3D; AG). HGF treatment reversed the percentage of cells in G0/G1 phase to control values (Fig. 3D; AG+HGF).

### HGF is a Survival Factor in Mammary Tumor Cells Derived from a Constitutively Activated ErbB2 Transgenic Mice

**Figure 4:**
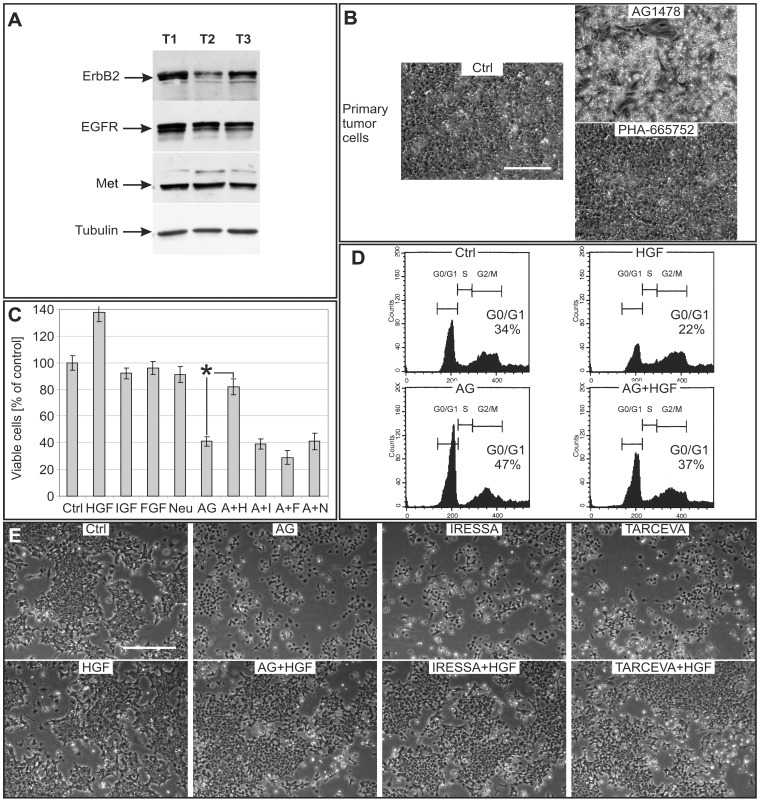
HGF is a survival factor of EGFR inhibited ErbB2 mammary tumor cells. A. Western-blot analysis of ErbB2, EGFR and Met expression in mammary tumors obtained from three separate transgenic ErbB2 mutated mice (see materials and methods). Tubulin was used as an internal loading control. **B.** Phase contrast images of primary cells from ErbB2 mammary tumors (bar  = 500 µm). Cells were grown in proliferation medium and either left untreated (Ctrl) or treated with AG1478 (250 nM) or PHA-665752 (250 nM) for 96 h. **C.** Viable cell count by trypan-blue exclusion staining at 48 h, of immortalized ErbB2 tumor cells treated with the indicated factors (HGF 10 ng/ml (H), IGF-I 100 ng/ml (I), b-FGF 20 ng/ml (F), Neuregulin 20 ng/ml (N); AG1478 250 nM (A)). Untreated control (Ctrl) was set to 100%. Columns, mean (n = 6); bars, S.E.M. *P<0.05. **D.** Cell cycle distribution examined by propidium iodide staining and FACS analysis of ErbB2 mammary tumor cells cultivated in growth medium and treated with the indicated factors for 16 h (HGF 10 ng/ml, AG1478 250 nM). **E.** Immortalized ErbB2 tumor cells cultivated in their respective growth medium for 48 h with the indicated factors (HGF 10 ng/ml; AG1478 250 nM; IRESSA 1 µM; TARCEVA 1 µM). Bar  = 500 µm.

We tested whether the effects discovered in normal mammary cells could be applied to cells isolated from a well described model of mutated-ErbB2 transgenic mice (MMTV-neu; [20]). We first visualized, by western-blot analysis, the presence of ErbB2, EGFR and Met in mammary tumor lysates obtained from different mice (Fig. 4A). Primary cells obtained from these tumors underwent cell death following 72 h treatment with AG1478 (250 nM) while PHA-665752 (250 nM) had no effect (Fig. 4B). In order to test whether AG1478-induced cell death could be recovered by Met activation, cells treated with AG1478 were supplemented with HGF. Cell viability reverted to control values, while supplementation of IGF-I, b-FGF and Neuregulin had no effect (Fig. 4C). Cell cycle analysis by propidium-iodide staining and flow cytometry showed an increase of cells in the G0/G1 phase after AG1478 addition (Fig. 4D, AG). Following HGF treatment cells reverted to values similar to untreated cells (Fig. 4D; AG+HGF). To confirm Met contribution to the recovery mechanism we used PHA-665752 together with AG and HGF. We found that in presence of this Met TKi, HGF lost its capacity to recover ErbB2 tumor cells from EGFR inactivation (Fig. S3A; A+P+H). To further confirm the specificity of AG1478, we treated these cells with two other EGFR inhibitors used in clinical protocols (Iressa and Tarceva; 1 µM). HGF retained the ability to recover ErbB2 tumor cells treated with Iressa and Tarceva (Fig. 4E and Fig. S3B). Therefore ErbB2 tumor cells obtained from this transgenic model are dependent on activated EGFR for growth and survival and can be rescued by Met activation.

### Met Phosphorylation Simultaneously Activates ERK1/2 and AKT when EGFR Signaling is Inhibited: Relative Importance of the ERK1/2 and AKT Pathways for Proliferation and 3D Morphogenesis

**Figure 5:**
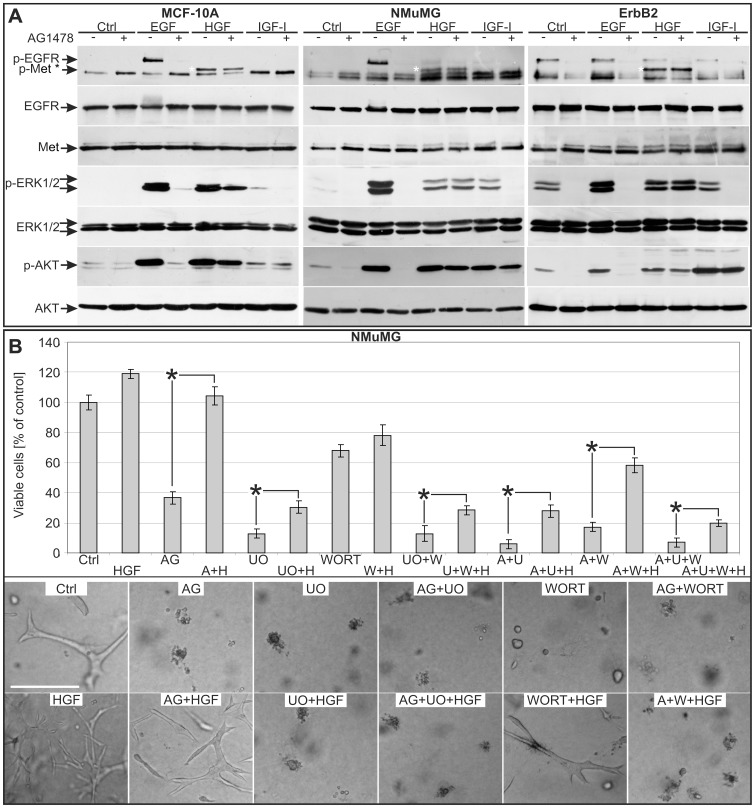
HGF activates Met, ERK1/2 and AKT in EGFR inhibited mammary cells: roles for ERK1/2 and AKT pathways. A. Western-blot analysis of the EGFR, Met, ERK1/2 and AKT phosphorylation status in MCF-10A and NMuMG cells and ErbB2 tumor cells cultivated in starving medium for 16 h and then treated with the RTK inhibitors AG1478 (250 nM, 1 h) before adding the indicated cytokines (10 min; EGF 10 ng/ml, HGF 10 ng/ml, IGF-I 100 ng/ml). **B.** Viable cell count by trypan-blue exclusion staining at 48 h of NMuMG cells cultivated in growth medium and the indicated inhibitors (AG/A = AG1478 250 nM; UO/U = UO126 15 µM; WORT/W = wortmannin 100 nM) in presence or absence of HGF 10 ng/ml (H). Untreated control (Ctrl) was set to 100%. %. Columns, mean (n = 6); bars, S.E.M. *P<0.05. **C.** Representative pictures of NMuMG cells grown in collagen gels for 4 days in growth medium with the indicated factors (concentrations as in B; bar  = 250 µm).

To understand the mechanism of recovery mediated by HGF, we performed western-blot analysis of the phosphorylated EGFR, Met and their downstream effectors ERK1/2 and AKT (Fig. 5A). For this purpose serum-starved MCF-10A and NMuMG cells were treated with EGF (10 ng/ml), HGF (20 ng/ml) or IGF-I (100 ng/ml) in the absence or presence of AG1478.

All growth factors activated both the ERK1/2 and the AKT pathways in the absence of EGFR inhibition (Fig. 5A, lanes 3, 5, 7). Following AG1478 treatment, HGF remained the only cytokine able to maintain both pathways active simultaneously, in agreement with the fact that phospho-Met is still present in EGFR inhibited cells (Fig. 5A, lane 6). Although IGF-I-activated phospho-AKT levels were unaffected by AG1478, phospho-ERK1/2 levels were abolished by EGFR inhibition (Fig. 5A, lane 8; see discussion). Therefore HGF-mediated rescue of proliferation and morphogenesis in mammary cells possibly needs simultaneous reactivation of both the ERK1/2 and AKT pathways.

Similar western blot analyses performed on serum-starved ErbB2 tumor cells showed basal phospho-EGFR, phospho-ERK1/2 and phospho-AKT levels that were abolished by AG1478 treatment. Analogously to normal mammary cells, HGF only had the ability to restore simultaneously ERK1/2 and AKT phosphorylation in presence of EGFR inhibition. Thus ErbB2-driven tumor cells possibly use a mechanism of recovery comparable to that found in non malignant NMuMG and MCF-10A cells (Fig. 5A, ErbB2). To further confirm the specificity of AG1478, western-blot experiments were repeated using Iressa (1 µM) in NMuMG and ErbB2-driven cells, with comparable results (Fig. S4A).

Since EGFR and Met seem to act through similar downstream effectors, we tested whether EGF and HGF simultaneous treatment could increase mammary cells growth, scatter and morphogenesis. Cells treated simultaneously with the two cytokines displayed an increase in confluence percentage (MCF-10A cells; Video S3 and Fig. S4B), scatter and morphogenesis (NMuMG cells; Figure S4C) relative to EGF only or HGF only treated cells.

We finally analyzed the relative importance of the ERK1/2 and AKT pathways during proliferation and morphogenesis in EGFR inhibited mammary cells (Fig. 5B). For this purpose we selectively inhibited ERK1/2 and/or AKT pathways with UO126 15 µM and Wortmannin 100 nM respectively. Such inhibitors were coupled with AG1478 treatment in order to understand whether HGF retained the ability to recover growth in mammary cells lacking these pathways. Inhibition of the ERK1/2 and PI3K-AKT pathways, separately and together with EGFR blockade, lead to a decrease in cell viability. Interestingly, under all inhibitory conditions, HGF increased cell numbers, albeit with different efficiencies according to treatment. This behaviour indicates that in the absence of any combination of EGFR/ERK1-2/AKT signaling, Met still enhances proliferation in 2D cultured cells (Fig. 5B upper panel and Fig. S5) probably activating alternative signaling pathways.

An analysis performed on NMuMG cells grown in 3D-collagen suspensions (Fig. 5B lower panel) gave us different results and showed that ERK1/2 signaling was always necessary to sustain viability: HGF supplementation could not restore viability in UO126 treated cells (UO; UO+HGF; AG+UO; AG+UO+HGF). On the other hand, inhibition of the PI3K-AKT signaling pathway stopped ductal elongation but did not kill cells (WORT). HGF addition restored growth and morphogenesis (WORT+HGF) in Wortmannin treated cells. Interestingly, when EGFR inhibited cells were simultaneously treated with Wortmannin, HGF could not act as a recovery agent (AG+WORT and A+W+HGF). These data indicate that in the absence of EGFR signaling the ERK1/2 and the PI3K-AKT pathways are both independently necessary for growth in a 3-dimensional matrix but not in a 2D proliferation assay.

## Discussion

Organ growth, homeostasis of normal tissues and tumor development may share common biological pathways. In this study we have addressed the relationship between EGFR and Met signaling pathways in normal mammary epithelial cells as well as in cells derived from a well described transgenic model of mammary tumorigenesis. We found that 2 out of 3 tested mammary epithelial cell lines, the murine NMuMG and the human MCF-10A cell lines, are dependent on EGFR activity in order to grow in a 2D proliferation assay and to survive in a 3D-morphogenesis assay. A fundamental role for the EGF receptor in mammary gland morphogenesis has been found during prepubertal growth in the stromal compartment of this organ [5], [21], but, as of now, there is no known function for this receptor in the epithelial compartment of the adult mammary gland. Here we described that EGFR is fundamental for growth and viability in specific non-tumorigenic mammary cell lines that have been obtained from adult mammary tissues. Impairment of the EGFR, but not Met signaling in these cell lines disrupted morphogenesis and induced complete cell death. MCF-10A dependence on EGFR may be due to these cells being cultured in an EGF containing medium. A possible explanation for EGFR addiction in NMuMG cells is that this cell line produces its own EGFR ligands. In fact we found high levels of amphiregulin mRNA expression in NMuMG cells (data not shown) possibly driving proliferation by an autocrine loop. Other mammary epithelial cells have been shown to produce and release EGFR ligands [22], therefore this mechanism of growth may be common to epithelial cells even in the adult mammary gland. The fact that BME-UV bovine mammary cells did not respond to EGFR inhibition is probably due to the fact that this cell line maintains the ERK1/2 and AKT pathways active under AG1478 treatment. One possible explanation is that, during the process of immortalization, inactivation of important tumor suppressors (like PTEN) have brought BME-UV cells to constitutively activate these fundamental signaling pathways. Another speculative hypothesis is that in bovines, that are not prone to develop mammary cancers, these pathways have constitutively higher phosphorylation levels even in non-tumorigenic cell lines. The difference in EGFR sensitivity found in cell lines, poses the question whether some mammary cells belonging to different compartments of the gland (e.g. basal or luminal) may have different requirements for growth. Expanding our research with other mammary cells lines of different origin may clarify this doubt. This fact is also important because tumors that arise from cells of a particular compartment may show different responses to specific inhibitors.

Although Met was dispensable for growth in all tested cell lines, HGF was the only cytokine able to recover proliferation and morphogenesis in EGFR inhibited cells. Some authors have described a direct role for HGF in the mammary gland [23], [24], but complete data that clarify the role that Met receptor plays *in vivo* are still lacking. Our laboratory is currently evaluating the role of Met by gene targeting with mammary specific promoters (MMTV-cre and Wap-cre), but coherently with the *in vitro* data here presented, we haven’t yet found a role for the HGF receptor in these transgenic models (manuscript in preparation). Interestingly, other growth factors (IGF-I, b-FGF and Neuregulin), that have been described as important mediators of mammary morphogenesis [10]–[14], [25], were unable to act as recovery agents in these cells. We cannot exclude that these factors may play an important role in cells derived from different mammary gland subpopulations or breast cancers. Recent evidence shows that EGFR may play a significant role in some HGF/Met mediated biological responses of mammary tumor and epithelial cells. In this system inhibition of the EGFR kinase with gefitinib or erlotinib abolished HGF induced proliferation and motility of some carcinoma cells [26]. On the contrary in our experimental settings Met was able to act as a recovery agent in EGFR inhibited cells and phospho-Met and phospho-EGFR seemed to be activated, at least in part, separately. Although we cannot rule out a certain degree of Met-EGFR transactivation in our system, most of the downstream signaling is evoked by an independent, EGFR-driven or Met-driven mechanism. The difference in the results may depend on the different cell lines that where used or their culture condition (we always used complete growth medium with serum and growth factors for most of the experiments). This result indicates how two specific pathways that originate from different receptors may drive similar cellular responses (e.g. viability or proliferation) independently of each other.

We also found that the synchronous activation of EGFR and Met increased growth, scatter and morphogenesis in NMuMG and MCF-10A cells. This effect, in which two separate tyrosine kinase receptors may act in a redundant and additive fashion to promote cell growth, is not unique to mammary cells. *In vivo*, during kidney morphogenesis, Met tissue specific knock-outs have reduced final nephron number. Crossing these mice with waved-2 spontaneous mutants, carrying a single point mutation that reduces EGFR kinase activity, affects ureteric bud branching as well as final glomerular number [17]. We recently showed that a similar mechanism of EGFR/Met redundancy happens also in the mammary gland [27].

ErbB2 is an important initiator of mammary tumorigenesis with more than 30% human breast cancers exhibiting ErbB2 amplification and overexpression [28]. EGFR is often a dimerization partner for ErbB2 both physiologically, during mammary gland development [13], [29], and pathologically in ErbB2 driven tumors [30]. We thus evaluated the effects of EGFR inactivation in a well described transgenic model of mammary tumorigenesis. This transgenic line expresses an MMTV promoter-driven rat-ErbB2 carrying a mutation that forces constitutive activation of the receptor [20]. All tumors obtained from ErbB2 mutated mice showed EGFR, ErbB2 and Met expression. EGFR inhibition caused death of primary and immortalized cells isolated from this tumor. Since EGFR inhibition by AG1478 at the concentration used in our assays is selective (IC50 3 nM; ErbB2 IC50 is >100 µM) the ErbB2 mutation possibly activates EGFR through hetero-dimerization. In fact we observed a decrease of both phospho-EGFR and phospho-ErbB2 (not shown) in ErbB2 tumor cells upon treatment with AG1478. Therefore we found that in this transgenic model, ErbB2 activation that drives tumor growth is coupled to EGFR kinase activity, which is fundamental for cell viability. HGF treatment rescued EGFR inhibited tumor cells, indicating that pathways similar to those present in the NMuMG and MCF-10A mammary cells may also be used by cancer cells to sustain their growth. The fact that tumor cells respond to HGF with increased proliferation and survival following EGFR inhibition has also been addressed in non-small cell lung cancer (NSCLC). A subset of NSCLC patients that develop resistance to EGFR inhibitors show increased HGF levels supporting a role for stromal derived HGF in promoting drug resistance [31], [32]. This type of resistance, in which normal receptor levels are accompanied by a higher expression of its respective growth factor in the surrounding stroma, may thus represent a mechanism used by tumor cells to escape RTKi treatment. Our findings on HGF-mediated recovery, extend the previously published results to mammary epithelial cells and ErbB2 mammary tumor cells, emphasizing the fact that molecular events present in non tumorigenic cells could be retained during tumor growth and become the basis for drug resistance.

One of the possible mechanisms underlying mammary cell recovery by HGF treatment following EGFR inactivation was the ability of this cytokine to activate both the ERK1/2 and the AKT pathways. Thus we investigated the relative importance of these two pathways in driving the resistance to EGFR inhibition. Our results show that HGF was the only cytokine with the capacity to activate the ERK1/2 and the AKT pathways independently of EGFR signaling. Confirming the observations made by other authors [33]–[35], who demonstrated that knockdown or blockade of EGFR blocked the IGF-I-induced MAPK (suggesting that IGF-I receptor may act upstream of EGFR) we found that IGF-I could not rescue the ERK1/2 pathway, but could rescue the AKT pathway in all EGFR inhibited cell lines. In line with its inability to rescue EGFR inhibited cells, basic-FGF was unable to activate neither the ERK1/2 nor the PI3K-AKT pathway in the NMuMG and MCF-10A cell lines (data not shown). Moreover both these cell lines did not increase proliferation following b-FGF or Neuregulin treatment. Thus, it is possible that b-FGF and Neuregulin were not functional in our cellular model, eventually for the lack of their respective receptors, identifying mammary cell lines with different growth factor requirements. Alternatively, some signaling molecules that are activated exclusively downstream EGFR or Met, but not by other growth factors, might contribute to the recovery mechanism. Breast tumor kinase (Brk) and ERK5 were recently shown to act as important mediators of migration in breast cancer cells downstream the EGF and the HGF receptors [36]. We are currently evaluating what is their role in our model.

The PI3K-AKT pathway plays a fundamental role in glioblastoma multiforme and small cell lung cancer. In these tumors redundant inputs provided by multiple RTKs drive signaling, growth and survival by maintaining a robust AKT activation [37], [38]. 2D proliferation assays showed that in the absence of EGFR inhibition, lack of activation of the ERK1/2 pathway led to a strong decrease in cell growth while impairment of the AKT pathway reduced cell numbers less efficiently, except in ErbB2 tumor cells. HGF treatment recovered cells in which ERK1/2 and/or AKT pathways were inhibited. When EGFR was simultaneously inactivated, HGF was still able to recover cell viability. Interestingly, addition of HGF recovered to almost control levels ErbB2 cells with EGFR and AKT simultaneously inhibited. Moreover, in the absence of EGFR signaling, activation of the AKT pathway alone, which is highly phosphorylated downstream of the IGF-I receptor, did not lead to growth recovery. Thus, in this 2D proliferation model, the ERK1/2 but not the AKT pathway, played a major, but not exclusive, role in proliferation.

3D-morphogenesis assays, which might best recall the physiological environment of mammary growth, provided us with interesting, although different, data. ERK1/2 were still the predominant kinases to sustain cell viability: inhibiting their activation by UO126 induced cell death and could not be recovered in the presence of HGF treatment. Therefore, in this assay, other parallel pathways (like the Brk/ERK5 pathway) cannot replace ERK1/2. On the other hand, AKT inhibition blocked basal branching and was recovered by Met activation, but when EGFR was coupled to AKT inhibition no recover was possible. Therefore, in these assays, HGF-mediated recovery following EGFR inhibition could only work if both the AKT and ERK1/2 kinases were simultaneously active and disrupting any of these signaling adaptors lead to cell death.

Despite the survival gains provided by anti-HER2 therapies, patients with advanced HER2+ breast cancer frequently display drug resistance. Often resistance occurs when mammary tumor cells alter some important cellular pathways, including hyperactivation of the PI3K-AKT or ERK pathways [39], [40]. Here we have shown that non-tumorigenic and tumorigenic mammary cells may use HGF to promote growth in the absence of EGFR signaling. Given that HGF, a cytokine produced by the mammary stroma, is induced by estrogen, a systemic hormone commonly produced during every estrous/menstrual cycle [41], this mechanism of resistance may represent an important escape to HER2 directed therapies.

The fact that initiation, maintenance and spread of cancer may require molecular events analogous to the ones that are active during physiological organ growth and remodelling is intriguing. The study of these pathways in the normal tissue may provide useful information for the treatment of the malignant reversion and may potentially affect the correct regimen in patients treated with tyrosine kinase inhibitors.

## Materials and Methods

### Reagents

All reagents, unless specified, were from Sigma–Aldrich (St. Louis, MO, USA); recombinant HGF, EGF, Insulin-Like Growth Factor I (IGF-I), Neuregulin and basic-Fibroblast Growth Factor (bFGF) were from Immunotools (Friesoythe, Germany) and were resuspended in PBS; AG1478, Gefitinib (Iressa), Erlotinib (Tarceva), UO126 and Wortmannin were from LC Laboratories (Woburn, MA, USA) and were resuspended in DMSO; PHA-665752 was from Tocris Bioscience (Ellisville, MI, USA); DC Protein Assay was from Bio-Rad Laboratories (Hercules, CA, USA); Hybond enhanced chemiluminescence (ECL) nitrocellulose membrane and Hyperfilm ECL were from GE Healthcare Bio-Sciences (Piscataway, NJ, USA); Super Signal West Pico Chemiluminescent Substrate was from Pierce (Rockford, IL, USA); Collagen type I was from BD Biosciences (Bedford, MA, USA). MTT colorimetric assay for cell proliferation was from Upstate (Temecula, CA, USA).

### Antibodies

Anti-Met mouse monoclonal antibody was from Zymed (1∶1000; South San Francisco, CA, USA); anti-EGFR rabbit polyclonal antibody was from Santa Cruz Biotechnology (1∶1000; SC-03; Santa Cruz, CA, USA); anti-α-tubulin (1∶5000) and anti-phospho-ERK1/2 (1∶5000; M 8159) mouse monoclonal antibodies were from Sigma–Aldrich; anti-ERK1/2 mouse monoclonal was from Upstate (1∶1000; MK12; Temecula, CA, USA); anti-phospho-Met rabbit polyclonal (tyr-1234/1235; 1∶1000), anti-phospho-AKT mouse monoclonal (ser-473; 1∶1000), anti-AKT rabbit polyclonal and anti-ErbB2 rabbit polyclonal antibodies were from Cell Signaling Technologies (Danvers, MA,USA).

### Cell Culture

MCF-10A non-tumorigenic human breast epithelial cells were purchased from the American Type Culture Collection (ATCC number CRL-10317; ATCC, Rockville, MD) and were grown in DMEM/F-12 media supplemented with cholera toxin (100 ng/ml), insulin (10 µg/ml), hydrocortisone (0.5 µg/ml), EGF (20 ng/ml) and 5% horse serum (Euroclone, Pero, Italy). NMuMG murine mammary epithelial cell line (ATCC number CRL-1636) was kindly provided by R. Montesano (University of Geneva Medical School, Switzerland) and cultured in DMEM supplemented with 10% FBS. BME-UV bovine mammary epithelial cell line was isolated and kindly provided by I. Politis (Agricultural University of Athens, Athens, Greece; [42]). BME-UV cells were grown in DMEM, supplemented with 10% FBS (Euroclone). Mouse liver extracts and ErbB2 primary cells and cell lines were obtained from lobular carcinomas arising in BALB/c transgenic mice harbouring a mutated rat Her-2/*neu* oncogene under the MMTV promoter [20]. Rat ErbB2 transgenic mice were bred by Biogem IRGS (Ariano Irpino, Italy), maintained in the transgenic unit of the Molecular Biotechnology Center (University of Torino) under a 12-hour light-dark cycle, and provided food and water ad libitum. Mice were treated in conformity with European laws and policies [43], and this study was approved by the Ethical Committee of the University of Torino. Briefly, fresh tissue (1∶2 w/v) obtained by tumor dissection and mincing, was added to a 1∶1 v/v mixture of DMEM/F12 medium supplemented with 2% w/v bovine serum albumin (BSA, Fraction V), 300 U/ml collagenase, 100 U/ml hyaluronidase, 100 U/ml penicillin, 100 µg/ml streptomycin (STEMCELL Technologies, Vancouver, BC, Canada) and placed in a shaking incubator at 37°C overnight. Epithelial cell aggregates obtained by centrifugation at 80 g for 30 seconds were washed in fresh DMEM/F12 medium for 3 times. Aggregates were incubated with a 0.5 mg/ml trypsin solution supplemented with 0.2 mg/ml EDTA followed by vigorous pipetting for 4 minutes and subsequent washing in Hank’s balanced salt solution (HBSS, STEMCELL Technologies) supplemented with 2% FBS. Cells were then treated for 3 min with 5 mg/ml dispase and 100 µg/ml DNAseI (STEMCELL Technologies) and passed through a 40 µm cell strainer (BD Biosciences, San Jose, CA, USA) to remove remaining cell aggregates. ErbB2 cells were then cultured in DMEM supplemented with 10% FBS, 10 000 U penicillin G (Euroclone) and 10 mg/ml streptomycin (Euroclone). Cells were maintained in a 5% CO_2_–water-saturated atmosphere and routinely passaged every 2–3 d by washing with PBS followed by trypsinization.

### Western Blot Analysis

For western-blot, all cell lines were seeded in six-well plates and allowed to grow to 50% confluence. If needed, cells were starved for 24 h in medium with no serum and 0.4% BSA. The indicated cytokines were added for the indicated time intervals. UO126 (15 µM), Wortmannin (100 nM), PHA-665752 (250 nM), AG1478 (250 nM), Iressa (500 nM) were resuspended in dimethyl sulfoxide (DMSO) in 10 mM stock solution and added 1h before lysis or the indicated treatments. Control samples were added with an equivalent amount of DMSO. Cells were washed with ice-cold PBS, lysed, and scraped in lysis buffer (20 mM Tris (pH 7.5), 150 mM NaCl, 1 mM EDTA, 1 mM EGTA, 1% Triton X-100, 1 mM h-glycerolphosphate) with Protease Inhibitor Cocktail (1∶100) and 1 mM sodium orthovanadate. Protein lysates (20 mg) were cleared of cellular debris by centrifugation at 4°C for 10 min at 12 000 g, quantified using DC Protein Assay, resolved in 10% SDS-PAGE gels, and transferred to Hybond-C Extra nitrocellulose membranes. After the transfer of proteins, the membranes were blocked at room temperature for 2 h with Tris-buffered saline (TBS, 10 mM Tris and 150 mM NaCl, pH 7.4) containing 10% BSA and then incubated overnight at 4°C with the appropriate primary antibodies. The membranes were washed six times for 5 min each in TBS–Tween and then incubated for 1 h at room temperature with HRP-conjugated secondary antibodies. The membranes were again washed six times in TBS–Tween and incubated for 5 min at room temperature with Super Signal West Pico ECL chemiluminescent substrate. The proteins were visualized by briefly exposing the membrane to an autoradiographic Hyperfilm ECL.

### MTT Cell Proliferation Assay

For MTT cell proliferation assay, MCF-10A, NMuMG and BME-UV cell lines were seeded in 96-well plates at low density and allowed to grow overnight. At 0 h the medium was removed and replaced with medium containing the indicated cytokines. Cell proliferation was evaluated at the indicated times using the MTT assay following the manufacturer’s instructions. Briefly, the intensity of the coloured compound formed (formazan dye) was quantified with an ELISA microplate reader. When MTT reagent was added the wells were subjected to further incubation for a period of 4 h to facilitate the reaction between mitochondrial dehydrogenase released from viable cells and tetrazolium salt of MTT reagent. The absorbance was measured at 550 nm, with the reference at 650 nm. Cell viability of cells at 0 h was set to 100%. Each experiment was repeated three times independently and in each experiment each treatment was performed with six replicate culture wells.

### 2D Growth Assay

Cells were seeded in six well plates at a density of 1×10^5^ cells per well and allowed to grow. After 24 h, the medium was removed and replaced with fresh medium containing the indicated factors and/or inhibitors. After 24–48 h cells were photographed with a Leica AF6000 LX (Leica Microsystems, Wetzlar, Germany) inverted microscope equipped with a Leica DFC350FX digital camera and a motorized stage controlled by the LAS AF software (Leica Microsystems, Wetzlar, Germany). Images of multiple fields were captured using the Tile Scan feature for automatic scanning (3×3 fields). Tile Scan image capture and merging was used to provide a single panoramic view at 50x magnification. For Video S3, MCF-10A cells cultivated in DMEM/F12 medium (5% horse serum without growth factors) for 16 h and photographed at 5 min intervals for 2 d. The video was edited using Wax (freeware video editing software http://www.debugmode.com/wax/; accessed August 2011).

### Trypan Blue Viability Assay

Cells were seeded in six well plates at a density of 1×10^5^ cells per well and allowed to grow. After 24 h, the medium was removed and replaced with medium containing 10% FBS and the indicated cytokines. Cell proliferation was evaluated after 48 h by trypsinization, resuspension in PBS, colouring with trypan blue (0.1%) and counting on Burker chambers. Nonviable, blue coloured cells, were excluded. Untreated control samples (Ctrl) were set to 100%. Each experiment was repeated three times independently, and in each experiment, each treatment was performed with two replicate culture wells.

### Cell Cycle Analysis

For cell cycle analysis cells were seeded in six-well plates in their respective growth medium and allowed to grow to 50% confluence then the indicated factors and/or inhibitors were added for 16 h. Cells were trypsinized and washed with PBS, then treated with RNAse (0.25 mg/ml) and stained with propidium iodide (50 mg/ml). The cell cycle distribution in G0/G1, S, and G2/M phases was calculated using the CellQuest program (BD Biosciences). Each experiment was repeated three times independently, and within each experiment, each treatment was performed with two replicate culture wells.

### Morphogenesis Assay

The morphogenesis assay was performed as described in [44]. Briefly, cells were trypsinized and washed with PBS. 5×10^3^ cells were embedded in 0.5 ml collagen gels. After the collagen solution gelled (37°C, 15 min), 1 mL of complete medium was slowly added to each well. After 48 h of growth the indicated factors were added and the cultures incubated at 37°C for 4 d and photographed with a Leica AF6000 LX inverted microscope. Frames taken from multiple Z-stacks were recomposed on a single picture using the extended depth of field freeware software, Combine Z from Alan Hadley (http://hadleyweb.pwp.blueyonder.co.uk/; accessed August 2012). For Video S1 and S2, NMuMG cells stably expressing EGFP, were embedded in collagen gels, cultivated for 3 d and then treated with the indicated factors/inhibitors. Cell were photographed at 30 min intervals for 2 d and the video was edited using Wax software.

### Statistical Analysis

Experimental data are presented as mean±sem. Statistical differences between treatments were calculated with one-way ANOVA using the Statgraphics package (STSC Inc., Rockville, MD, USA). When significant differences were found, means were compared by an unpaired Student’s t-test.

## Supporting Information

Figure S1 **Western-blot analysis of the EGFR and Met phosphorylation.** MCF-10A cells were cultivated in starving medium for 16 h, then treated with the indicated RTK inhibitors for 45 min (PHA-665752, PHA, Met inhibitor; AG1478, AG, EGFR inhibitor; 250 nM) and finally with EGF or HGF for 10 min (10 ng/ml).
(TIF)

Figure S2 **Viable cell count by trypan-blue exclusion staining at 48 h of MCF-10A and NMuMG cells cultivated in growth medium and the indicated inhibitors (AG/A = AG1478 250 nM; PHA/P = PHA-675752 250 nM) in presence or absence of HGF 10 ng/ml (H). Untreated control (Ctrl) was set to 100%.** Columns, mean (n = 6); bars, S.E.M. *P<0.05.
(TIF)

Figure S3 **A/B Viable cell count by trypan-blue exclusion staining at 48 h of ErbB2 tumor cells cultivated in growth medium and the indicated inhibitors (AG/A = AG1478 250 nM; PHA/P = PHA-675752 250 nM; IRE/I = IRESSA 1 µM; TAR/T = TARCEVA 1 µM) in presence or absence of HGF 10 ng/ml (H).** Untreated control (Ctrl) was set to 100%. Columns, mean (n = 6); bars, S.E.M. *P<0.05.
(TIF)

Figure S4 **A. ERK1/2 and AKT phosphorylation in starved NMuMG cells and ErbB2 tumor cells treated with Iressa and the indicated cytokines.** **B.** % confluence calculated from the experiment shown in Video S3. **C.** 2D-growth (upper fields) and 3D-morphogenesis (lower fields) of NMuMG cells treated with the indicated factors (10 ng/ml).
(TIF)

Figure S5 **Viable cell count by trypan-blue exclusion staining at 48 h of MCF-10A and ErbB2 tumor cells cultivated in growth medium and the indicated inhibitors (AG/A = AG1478 250 nM; UO/U = UO126 15 µM; WORT/W = Wortmannin 100 nM) in presence or absence of HGF 10 ng/ml (H).** Untreated control (Ctrl) was set to 100%. %. Columns, mean (n = 6); bars, S.E.M. *P<0.05.
(TIF)

Video S1 **NMuMG cells stably expressing EGFP were grown in collagen for 2 days.** Cells were then treated with AG1478 250 nM and time lapse acquisition (48 h; 100x magnification) was made using a computer controlled Leica AF6000LX inverted microscope equipped with a motorized stage controlled by the LAS AF software.
(MOV)

Video S2 **NMuMG cells cultured as in Video S1 were treated with the indicated factors (HGF 10 ng/ml; AG1478 250 nM) and time lapse acquisition was made as in Video S1.**
(MOV)

Video S3 **MCF-10A cells were starved overnight in medium containing 5% horse serum and then treated with the indicated factors (10 ng/ml).** Time lapse acquisition (6 h; 50×magnification) was made using a computer controlled Leica AF6000LX inverted microscope equipped with a motorized stage.
(MOV)
