# Supplementary figures and images for: Met Receptor Acts Uniquely for Survival and Morphogenesis of EGFR-Dependent Normal Mammary Epithelial and Cancer Cells
Source: PLoS One. 2012 Sep 13;7(9):e44982. doi: 10.1371/journal.pone.0044982 (PMC3441651; doi:10.1371/journal.pone.0044982)

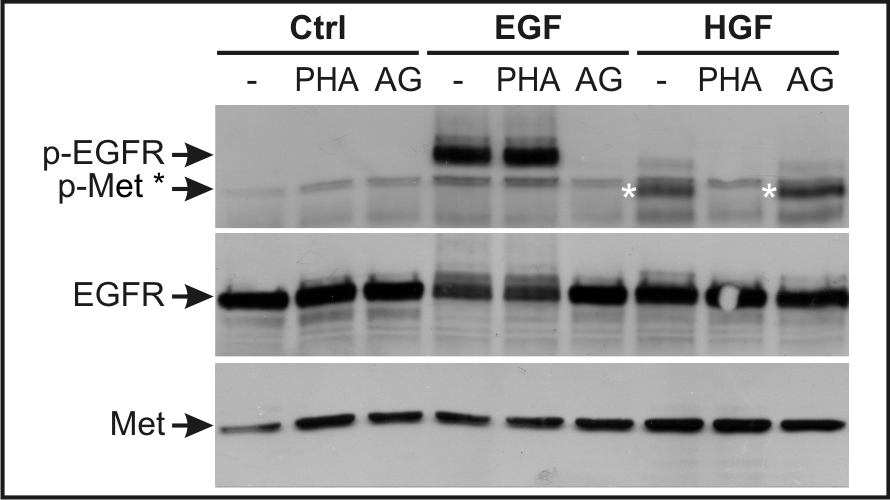

Supplement: Figure S1 — Western-blot analysis of the EGFR and Met phosphorylation. MCF-10A cells were cultivated in starving medium for 16 h, then treated with the indicated RTK inhibitors for 45 min (PHA-665752, PHA, Met inhibitor; AG1478, AG, EGFR inhibitor; 250 nM) and finally with EGF or HGF for 10 min (10 ng/ml). (TIF) [file pone.0044982.s001.tif]

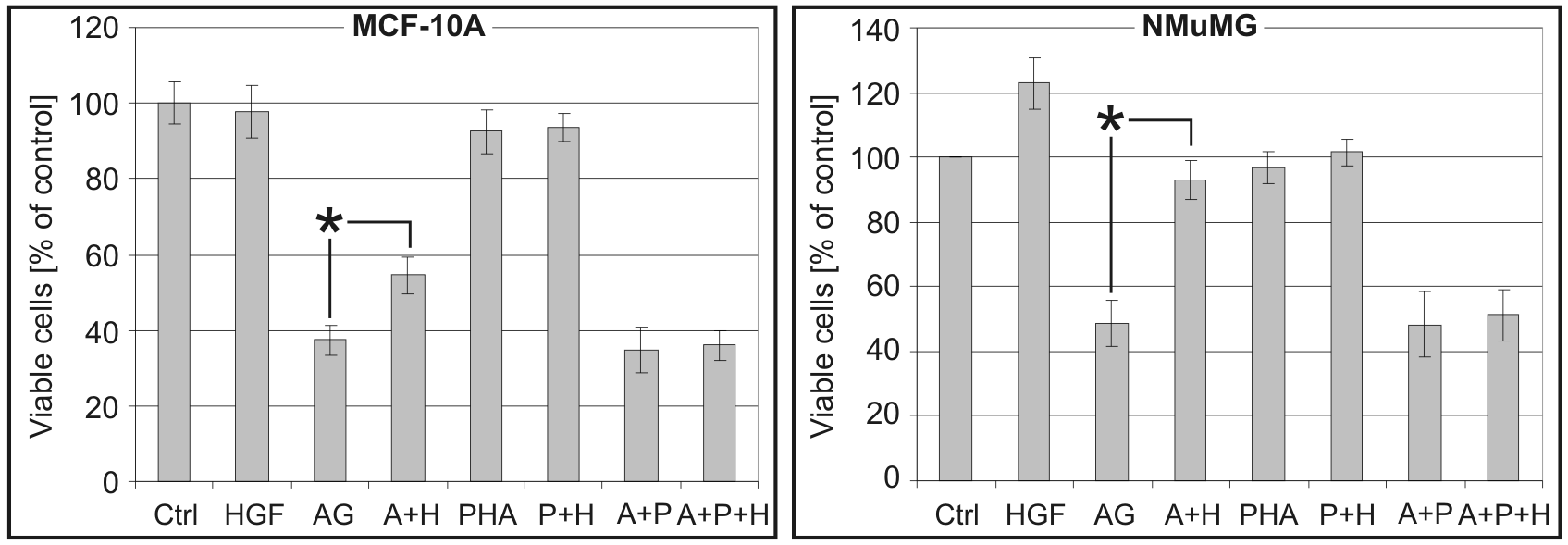

Supplement: Figure S2 — Viable cell count by trypan-blue exclusion staining at 48 h of MCF-10A and NMuMG cells cultivated in growth medium and the indicated inhibitors (AG/A = AG1478 250 nM; PHA/P = PHA-675752 250 nM) in presence or absence of HGF 10 ng/ml (H). Untreated control (Ctrl) was set to 100%. Columns, mean (n = 6); bars, S.E.M. *P<0.05. (TIF) [file pone.0044982.s002.tif]

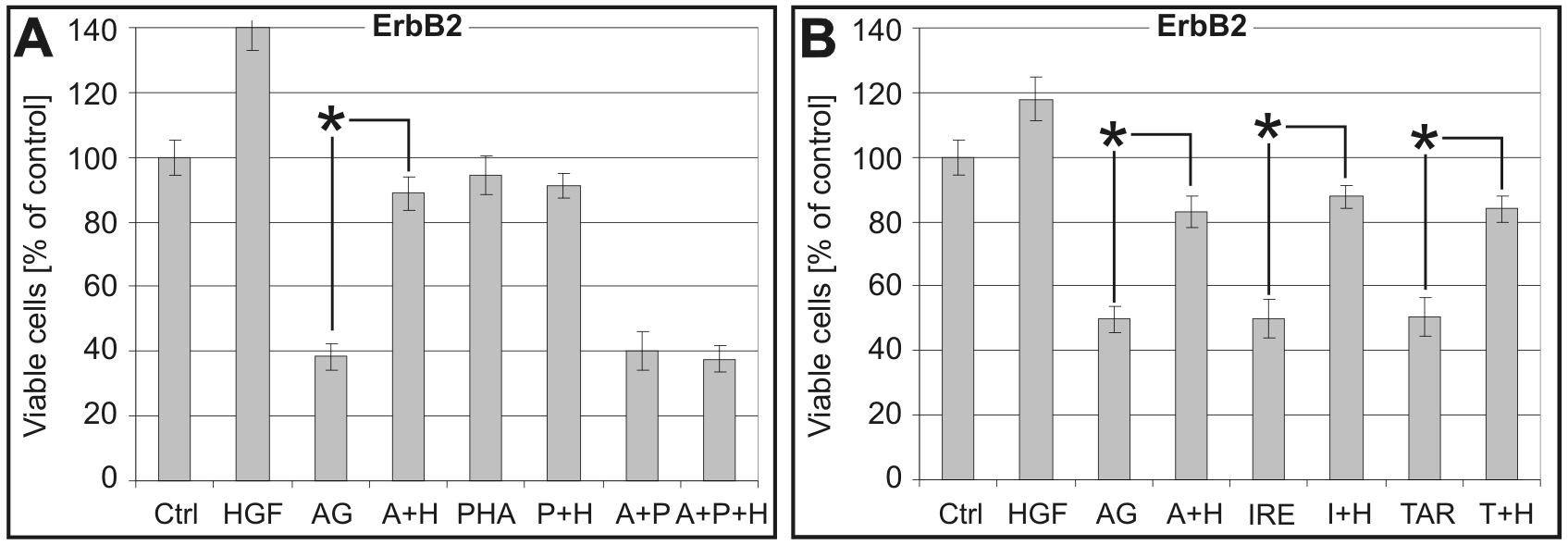

Supplement: Figure S3 — A/B Viable cell count by trypan-blue exclusion staining at 48 h of ErbB2 tumor cells cultivated in growth medium and the indicated inhibitors (AG/A = AG1478 250 nM; PHA/P = PHA-675752 250 nM; IRE/I = IRESSA 1 µM; TAR/T = TARCEVA 1 µM) in presence or absence of HGF 10 ng/ml (H). Untreated control (Ctrl) was set to 100%. Columns, mean (n = 6); bars, S.E.M. *P<0.05. (TIF) [file pone.0044982.s003.tif]

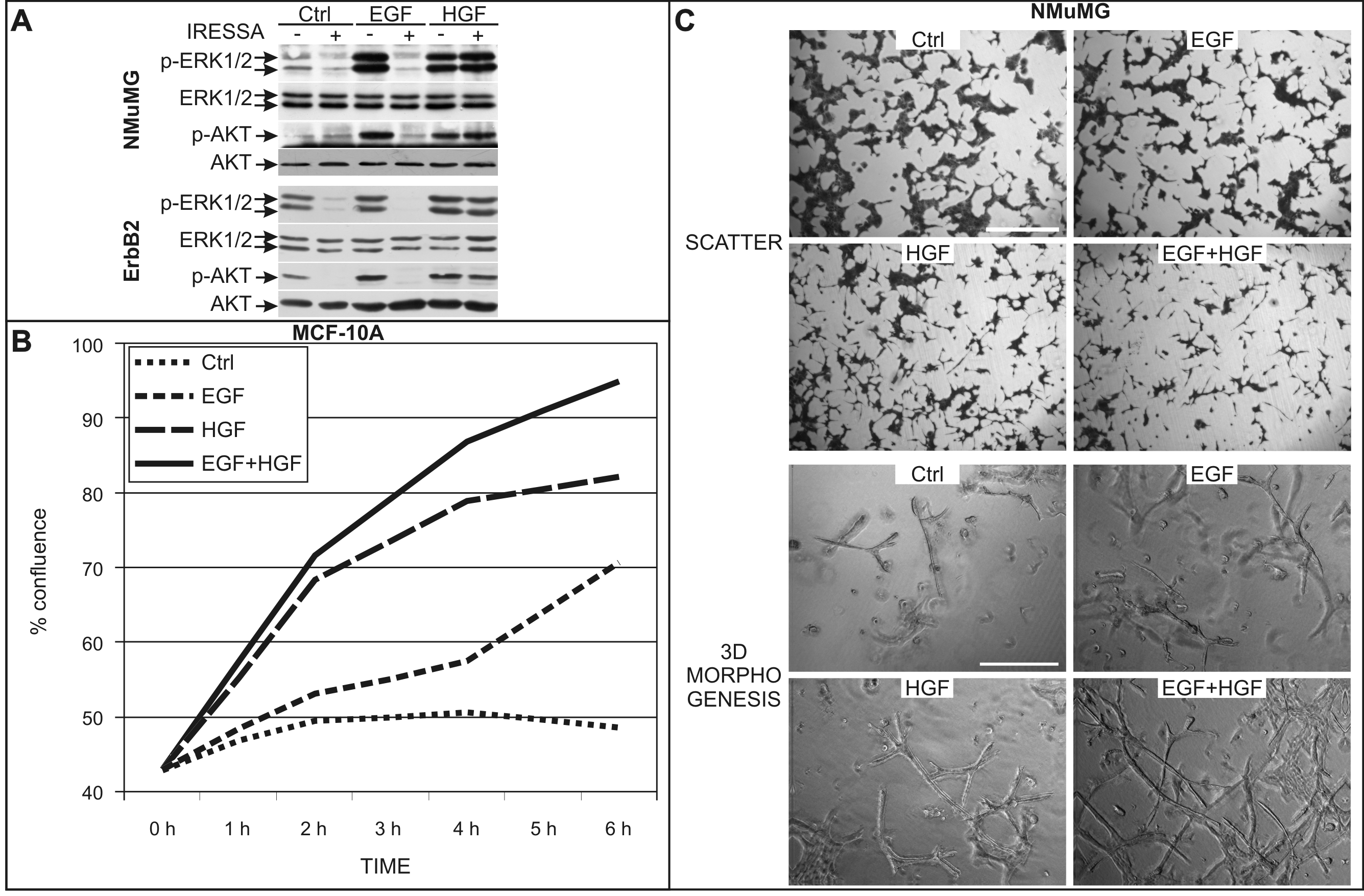

Supplement: Figure S4 — A. ERK1/2 and AKT phosphorylation in starved NMuMG cells and ErbB2 tumor cells treated with Iressa and the indicated cytokines. B. % confluence calculated from the experiment shown in Video S3. C. 2D-growth (upper fields) and 3D-morphogenesis (lower fields) of NMuMG cells treated with the indicated factors (10 ng/ml). (TIF) [file pone.0044982.s004.tif]

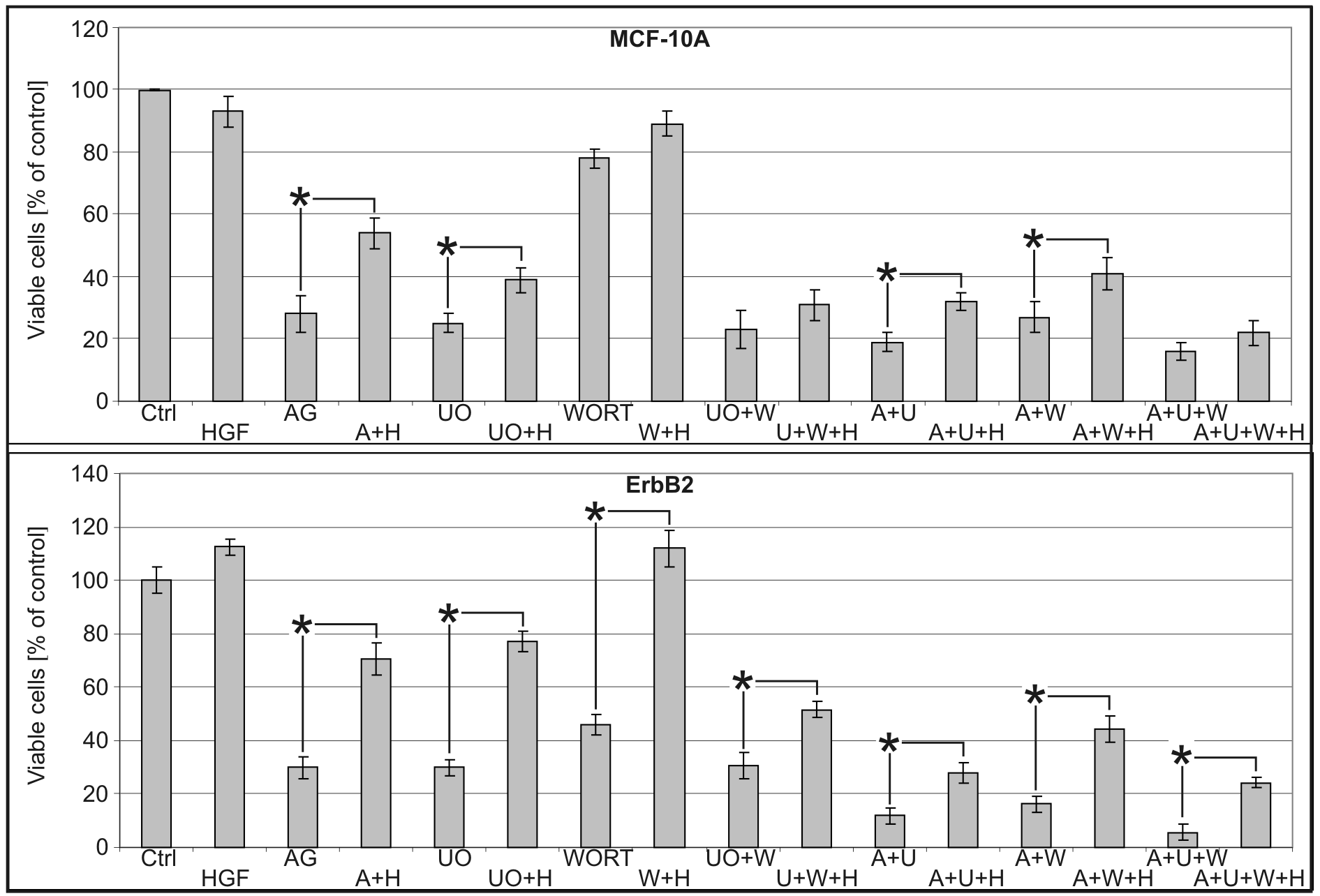

Supplement: Figure S5 — Viable cell count by trypan-blue exclusion staining at 48 h of MCF-10A and ErbB2 tumor cells cultivated in growth medium and the indicated inhibitors (AG/A = AG1478 250 nM; UO/U = UO126 15 µM; WORT/W = Wortmannin 100 nM) in presence or absence of HGF 10 ng/ml (H). Untreated control (Ctrl) was set to 100%. %. Columns, mean (n = 6); bars, S.E.M. *P<0.05. (TIF) [file pone.0044982.s005.tif]
